# An Anti-Stigma Course for Occupational Therapy Students in Taiwan: Development and Pilot Testing

**DOI:** 10.3390/ijerph17155599

**Published:** 2020-08-03

**Authors:** Hui-Ing Ma, Chu-En Hsieh

**Affiliations:** 1 Department of Occupational Therapy, College of Medicine, National Cheng Kung University, Tainan 700, Taiwan; 9804018@gs.ncku.edu.tw; 2 Institute of Allied Health Sciences, College of Medicine, National Cheng Kung University, Tainan 700, Taiwan

**Keywords:** anti-stigma, medical education, occupational therapy students

## Abstract

Attitudes of healthcare professionals towards people with disorders/disabilities are important for the development of therapeutic relationships, as well as to the evaluation and intervention processes. Therefore, it is critical to be aware and reduce stigmatizing attitudes in future healthcare professionals. An 18-week anti-stigma course was developed for occupational therapy students based on literature review and focus group interview. The course consisted of three components, including social contact, roleplaying, and critical reflection strategies. A quasi-experimental design was implemented to evaluate participants at three time points (i.e., pre-test, post-test, and one year after completion) using the Social Distance Scale and several questionnaires (i.e., stigmatising attitudes towards mental illness, physical disabilities, and children with emotional behavioural disorders). A total of 16 students completed the course and had significantly decreased social distance and stigmatising attitudes towards mental illness and emotional behavioural disorders in the post-test. These decreases remained one year later. The results support the provision of an anti-stigma course for occupational therapy students to reduce stigmatising attitudes. Future research should extend the anti-stigma course to occupational therapy students at other universities to increase both the sample size and overall generalisability.

## 1. Introduction

Stigma can be understood as incorrect understandings, negative attitudes, and discriminatory behaviours towards people with devalued characteristics [1]. Disorders and disabilities are often stigmatised by members of the general public, including healthcare practitioners [2,3]. In fact, some patients have reported stigma-related experiences when interacting with healthcare professionals [4]. These experiences are likely to aggravate existing feelings of rejection and incompetence, thus hindering these individuals from seeking and participating in treatments [5].

Students entering the healthcare profession may share public stigma rooted in our sociocultural system [6]. However, medical education has been found to both reduce and aggravate stigma [7]. Conventional education on mental illness did not reduce stigmatising attitudes or behaviours [8,9]. Therefore, educational intervention targeting stigma awareness and reduction is needed [10].

Many anti-stigma programs have recently found that contact-based strategies can effectively reduce mental-illness related stigma held by nursing, pharmacy, and medical students [11,12,13]. Following this line of evidence, researchers in Canada developed the Process Model for Successful Anti-Stigma Programming for Students in Healthcare [14,15]. The model identifies several key learning needs, such as understanding the roots of stigma held by healthcare providers and emphasising the importance of including multiple forms or points of social contact. However, it may not be easy to implement some of these elements in Eastern cultures due to the difficulty of finding people with disorders/disabilities who are willing to disclose their stories. Appropriate complementary strategies should thus be considered. In this regard, critical reflection may be a useful strategy for enhancing stigma awareness and reduction. Some of the beliefs and propositions that underlie stigmatising attitudes may be uncritically acquired during childhood as a result of the socialisation process [8]. New perspectives can thus be induced through critical reflection on these problematic hidden beliefs and attitudes [16,17,18]. Given this provision, students are likely to become critically aware of implicit and explicit stigma in society. This enables them to reformulate their assumptions in order to adopt more inclusive and integrative perspectives, which ultimately leads to corrective action based on better understanding [19,20].

Many of the anti-stigma programs aimed at healthcare students are designed to reduce stigma about mental illness [11,12,13,21,22]. However, other disorders and disabilities may also be stigmatised. For instance, children with emotional behavioural disorders (EBD) are usually considered troublemakers, and their parents are blamed for their children’s problems. People with physical disabilities tend to be regarded as a burden on their family and society. These populations usually require healthcare and rehabilitation services to assist them in adapting to their difficulties and achieving their full potential. Therefore, attitudes of healthcare professionals towards these people and their families in this process play a critical role in their motivation and intention to become involved in therapy [23]. This makes it imperative to ensure that anti-stigma programs address a variety of disorders and disabilities. Therefore, the purpose of this study was to develop and pilot test an anti-stigma course based on contact-based strategy and critical reflection for occupational therapy (OT) students. We hypothesised that the course would reduce stigmatising attitudes held by participating future professionals.

## 2. Materials and Methods

### 2.1. Study Design and Participant Recruitment

A quasi-experimental design was used to assess the outcomes of the anti-stigma course. Specifically, the course was set as a two-credit elective that lasted 18 weeks at National Cheng Kung University in Taiwan during the spring semester of 2018. A total of 27 second-year OT college students initially registered, but 11 of these later dropped the course. Questionnaires on social distance and stigmatising attitudes were distributed on the first day, thus constituting the pre-test assessment (*n* = 27), while the post-test was conducted on the last day of the course using the same materials (*n* = 16). One year later, questionnaires were distributed a third time to conduct a follow-up assessment; participants included students who completed the course (*n* = 15), those who dropped the course (*n* = 10), and those who had not yet registered (*n* = 10). Data from two students (one who completed the course and one who dropped) were missing in the one-year follow-up due to loss of contact. Only data from students who completed and dropped the course were analysed in this study. All participants gave their informed consent for inclusion before they participated in the study. The study was conducted in accordance with the Declaration of Helsinki, and the protocol was approved by the Human Research Ethics Committee at National Cheng Kung University (NCKU HREC-E-105-300-2).

### 2.2. Measures

Because the OT practice is primarily concerned with people with mental illness, children with EBD, and people with physical or intellectual disabilities, we used the Social Distance Scale and questionnaires on stigmatising attitudes towards these populations. A 6-point Likert scale was used for all measures, with 1 indicating “strongly disagree” and 6 indicating “strongly agree”. In this study, we presented the average scores for the items on each questionnaire (i.e., a possible range of 1 to 6).

#### 2.2.1. Social Distance Scale

The Social Distance Scale was used to measure one’s willingness to participate in social contacts entailing varying degrees of closeness with people with mental illness [24]. It contains 7 items, with higher scores indicating greater preferences for social distance. Internal consistency was supported with α values of 0.80 and 0.78 for schizophrenia and depression, respectively [25].

#### 2.2.2. Questionnaires on Stigmatising Attitudes

Questionnaires on stigmatising attitudes towards mental illness, children with EBD, and disabilities were developed during the first year of this three-year prospective project, which was aimed at developing an anti-stigma program for OT students [26]. Specifically, the Questionnaire on Stigmatising Attitudes Towards Mental Illness consisted of 16 items across four subscales (i.e., deviant behaviour, social isolation, negative stereotypes, and self-stigma), the Questionnaire on Stigmatising Attitudes Towards Children with EBD consisted of 14 items across three subscales (i.e., rejective attitude, negative stereotypes, and deviant behaviour), and the Questionnaire on Stigmatising Attitudes Towards Disabilities consisted of six items across two subscales (i.e., negative stereotypes and pessimistic expectations). For all questionnaires, higher scores indicated greater negative stigmatising attitudes. Psychometric testing suggested that the questionnaires were of satisfactory internal consistency (Cronbach’s α = 0.89, 0.86, and 0.71 for mental illness, EBD, and disabilities, respectively).

### 2.3. Anti-Stigma Course

**Figure 1:**
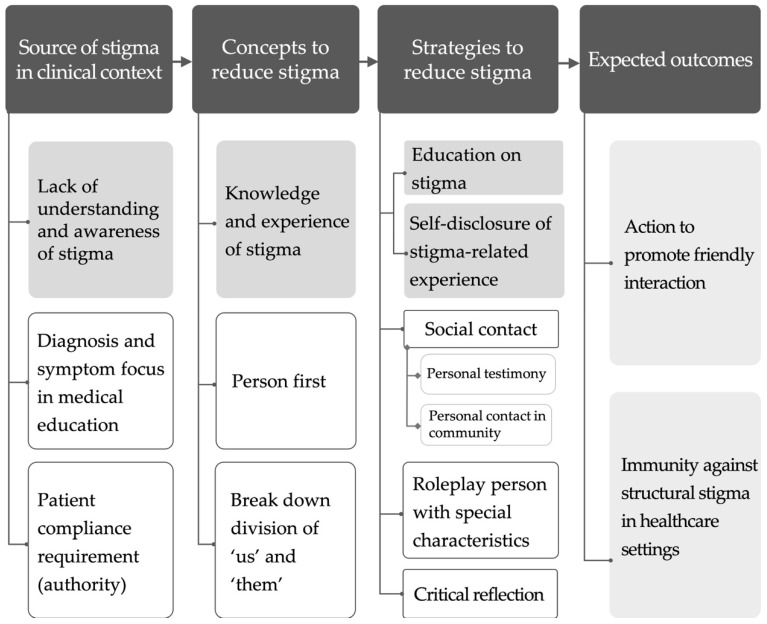
Themes constructed from focus-group interview transcript to develop the anti-stigma course for occupational therapy students.

The anti-stigma course was developed following a focus group interview. Specifically, four experts with clinical, teaching, or research experiences related to stigma agreed to participate in this process. A preliminary course framework was first drafted based on the Process Model for Successful Anti-Stigma Programming for Students in Healthcare [14], which served as the background for discussion. We then presented the following issues during the focus group interview: (1) how to cognise stigmas, (2) how to help students become aware of their implicit stigmas, and (3) how to design a course that stimulates student reflections and critical thinking about stigmatising attitudes and behaviours. A protocol analysis was then conducted on data from the interview transcriptions [27]. The four following themes were thus constructed: (1) sources of stigma in the clinical context, (2) concepts for reducing stigma, (3) strategies for reducing stigma, and (4) expected outcomes (Figure 1).

Based on the results of the focus group interview, we developed a course framework by proceeding from knowledge to experience and then to action (Table 1). Here, multi-mode teaching approaches were implemented. In addition to receiving lectures and watching videos/movies, students were required to participate in experiential activities involving social contact, roleplaying, critical reflection (assignments 1 and 2), and an action project (assignment 3). We also used The Human Library ^®^ to recruit a person living with chronic schizophrenia to share his story and hold class discussions. The three major group assignments were (1) accompanying a person with disability in the community, (2) roleplaying a person with special characteristics and their caregivers in public, and (3) conducting an action project to promote friendly interaction with people with disorders/disabilities. Students were also required to write reflection essays based on the first two assignments. Guidelines were provided to help students critically reflect, including (1) discussing the processes and assumptions underlying actions and thoughts, (2) analysing the roles of associated emotions and relevant past experiences, (3) considering the experience from multiple perspectives, (4) stating the lessons learned, and (5) establishing a plan to improve future behaviours and outcomes [28].

### 2.4. Data Analyses

All statistical analyses were conducted using the IBM SPSS 17.0 software (SPSS Inc., Chicago, IL, USA). Due to the small sample size, non-parametric tests were used. The Friedman test was used to compare pre-test, post-test, and one-year follow-up scores. Post hoc analyses were applied if the overall Friedman test result was statistically significant [29]. In addition, Mann–Whitney U tests were conducted to compare students who completed the course with those who dropped the course in regard to the pre-test scores and the score changes between the pre-test and one-year follow-up.

## 3. Results

### 3.1. Participant Characteristics

Participant characteristics are available in Table 2. As shown, no significant differences were found between students who completed the course and those who dropped the course.

### 3.2. Evaluation Results

For students who completed the course, pre-test, post-test, and follow-up scores for the Social Distance Scale are presented in Table 3, and scores for the stigmatising attitudes questionnaires are presented in Table 4. As shown, scores were significantly lower for the post-test and follow-up when compared with pre-test results for most items on the Social Distance Scale and the subscales for stigmatising attitudes towards mental illness and children with EBD. These results suggest that social distance and stigmatising attitudes decreased after the course. Notably, these decreases were found to remain one year after course completion.

Finally, Table 5 shows a comparison of pre-test and follow-up score changes between students who completed the course and those who dropped the course. As shown, there were significant differences in five out of seven items on the Social Distance Scale. These results suggest that students who completed the course had significantly greater decreases in social distance towards persons with mental illness than those who dropped the course. However, we also noted that students who completed the course had higher pre-test scores in the Social Distance Scale (item 2: *Z* = 2.83, *p* = 0.007; item 4: *Z* = 2.89, *p* = 0.007; item 5: *Z* = 2.10, *p* = 0.048) than those who dropped the course.

## 4. Discussion

OTs interact with children and adults with various disorders and disabilities in their professional practice. While unique stigmas may be associated with different clinical populations, there are also commonalities between types. For that reason, this study’s anti-stigma course began with general knowledge about stigma and then used experiential activities (e.g., social contact and roleplaying) and critical reflection to expand the focus to not only mental illness but also various disorders/disabilities and their families/friends (courtesy stigma). Our hypothesis was generally supported. By using the Social Distance Scale and questionnaires on stigmatising attitudes, we demonstrated that the course significantly reduced the stigmatising attitudes that OT students held towards mental illness and EBD. Moreover, we found that these effects remained one year later. Further, the students who completed the course showed greater decreases in social distance towards mental illness from pre-test to the one-year follow-up when compared with students who dropped the course.

We believe that the combination of social contact, roleplaying, and critical reflection strategies in this course has contributed to the decrease in stigmatising attitudes exhibited by students through their test results. In regard to the social contact, students heard a personal testimony from a person recruited through The Human Library ^®^ and accompanied individuals with disabilities to jointly perform activities chosen by those individuals. Therefore, students had opportunities to fully interact with them as full human beings rather than merely viewing them in the clinical context. We also observed that the individuals with disabilities chose activities that they were familiar with and competent in (e.g., adapted triathlon), thus demonstrating their capabilities to the students. Based on their reflection essays, we found that this type of personal and positive contact helped counteract previous stereotypes and prejudices held against people with disabilities.

For the second assignment, we observed that many students experienced heightened stress and anxiety as the time of roleplaying in public approached. Specifically, they questioned the assignment’s rationale and wondered if it is dishonestly masquerading in public. For that reason, we had several discussions about the rationale and provided a fact sheet that could be handed to anyone in public wishing for an explanation. However, some students still dropped the course without specifying why. We speculate that the stress and anxiety associated with publicly roleplaying a person with special characteristics (especially one with mental illness) may have somehow reflected the difficulty associated with breaking the division between “us” and “them” [30]. That is, we are normal people, so we cannot and will not behave abnormally (like them). However, is it really true that we cannot or will not be abnormal one day?

Critical reflection aids in deeper explorations of the given issues. By reflecting on their experiences of roleplaying as persons with disabilities or caregivers, students became aware of their implicit self-stigmatisation and courtesy stigma. Such stigma-related feelings may be traced to shared public stigma rooted in society. The roleplaying experiences may have also partly broken down the us–them division, as some students reported learning that stigma was not an issue confined to others but may one day occur to them or their families and friends.

Finally, we observed that self-directed learning occurred as students worked on their action projects. For example, one group of students developed a boardgame to enhance the understanding of stigma issues related to various disorders/disabilities. They then played the game with passers-by at the university and on the street. The process of generating questions for the boardgame and interacting with passers-by increased their knowledge and helped clarify their own initial opinions. By providing opportunities to “do something”, we believe that the action project may have consolidated new perspectives that students gained during earlier experiential activities and critical reflection.

Regarding the difference in change scores between students who completed the course and those who dropped it, we found significantly greater decrease in stigmatising attitudes among the former group. We also noted that students who completed the course had higher pre-test scores in the Social Distance Scale (items 2, 4, and 5) than those who dropped the course. A previous study also reported greater decreases in stigmatising attitudes among students who initially had higher stigmatising attitudes [31]. These findings suggest that, although a strong stigmatising attitude may at first appear negative and harsh, it may actually indicate amenability. It is therefore necessary to understand that mere protest against stigma can have adverse effects, including rebound behaviours that result in increased bias [19,20]. It is thus important to provide a safe and non-threatening environment so that students can share their experiences and thoughts without being criticised. In addition, significant intergroup differences were only found in the Social Distance Scale, not in the other questionnaires. This may be because the Social Distance Scale is less subject to influences stemming from the desire for positive self-presentation than the others [32,33,34]. Future research may adapt the Social Distance Scale to measure implicit stigma towards other populations.

The drop rate of this anti-stigma course is higher than the other electives in our department. Based on our observation, the high drop rate may be attributed to several factors. First, this is a new course as compared to the other electives that have been offered for years. While students can gather information about the other electives from former students, topics and assignments of this anti-stigma course are new to students, and they may find this is not what they expected when registering for the course. Second, in contrast to the other electives that are more knowledge- and skill-oriented and related to the content area of the certification examination, this anti-stigma course focuses on critical reflection on attitudes, which is not required for the certification examination. Therefore, students may drop this course if they feel overloaded, especially given that the reflection essays and novel experiential activities are challenging and frustrating for some students.

This study has some limitations. First, the anti-stigma course was offered as an elective in the OT department, thus making it impossible for us to randomly assign students to a control group. Such a quasi-experimental design limits our ability to determine causality. In addition, given that our OT department only recruits around 30 students each year, our sample size is limited. This restricts our power to detect significant differences and establish generalisability. Future research may extend the anti-stigma course to OT departments at other universities to increase both the sample size and overall generalisability. In addition, future research may include outcome measures of implicit stigma [35] and discriminatory behaviours [36] to obtain a comprehensive picture of the effectiveness of anti-stigma program.

Stigma operates at various levels [37]. At the intrapersonal level, stigma is associated with compromised psychological well-being of not only people with disorders/disabilities [38] but also healthcare professionals [39]. At the interpersonal level, stigma may adversely affect the development of therapeutic rapport, and the evaluation and intervention processes, and thus further contribute to healthcare disparities at the structural level [35]. Therefore, the anti-stigma program is imperative, and its implications can be manifold. In this study, we demonstrated the development and implementation of an effective anti-stigma course for OT students. Continual refinements of the program, extension to other healthcare professions, and multi-dimensional outcome evaluations are needed in future work, with a long-term goal to enhance the psychological well-being of both people with disorders/disabilities and healthcare professionals, to deliver quality healthcare, and to pursue health equity [40].

## 5. Conclusions

This is the first study to develop and pilot-test an anti-stigma course for OT students. We found that students who completed the course had significant decreases in stigmatising attitudes towards mental illness and EBD. Importantly, these decreases persisted based on a one-year follow-up. Our findings thus suggest that a combination of social contact, roleplaying, and critical reflection strategies may decrease stigmatising attitudes. This study demonstrates the development and implementation of an effective anti-stigma course for healthcare students. Continual refinements and evaluations are still needed to strengthen the course while further determining its long-term effects.

## Figures and Tables

**Table 1:** Course framework.

Stage	Objectives	Activities/Assignments
Knowledge (cognition)	Understand the concept of stigma Understand the phenomena and influence of stigma in healthcare settings Identify social discrimination	Lecture Watch videos Movie “Mad World” In-class discussion
Experience (reflection)	Be aware of one’s own implicit stigma Be aware of one’s own stereotypes, prejudices, and discriminations against people with disorders/disabilities Experience the stigma-related feelings of people with disabilities and their caregivers	Accompanying a person with disability Roleplay a person with special characteristics ad his/her caregivers The human library ^®^
Action (practice)	Enhance friendly interaction between general society and persons with disorders/disabilities	Action project

**Table 2:** Participant characteristics.

	Pre-Test		One-Year Follow-Up	
Characteristics	Students Who Completed the Course (*n* = 16)	Students Who Dropped the Course (*n* = 11)	Students Who Completed the Course (*n* = 15) ^2^	Students Who Dropped the Course (*n* = 10) ^2^
Women (%)	8 (50%)	10 (91%)	8 (53%)	9 (90%)
Age, years (M ± SD)	20.31 ± 1.195	19.91 ± 0.831	21 ± 1.254	21 ± 0.667
SES ^1^ (M ± SD)	3.44 ± 0.727	3.27 ± 0.467	3.27 ± 0.594	3.20 ± 0.422
Have suffered from physical or mental illness				
Yes (%)	0	1 (9%)	1 (7%)	1 (10%)
Have family members or friends with disabilities				
Yes (%)	8 (50%)	4 (36%)	7 (47%)	3 (30%)

^1^ SES = socioeconomic status, ranging from 1 (low) to 5 (high). ^2^ Data from two students (one completed, and one dropped the course) were missing due to loss of contact.

**Table 3:** The results of Friedman test on the pre-test, post-test, and follow-up scores for the Social Distance Scale among students who completed the course (*n* = 15).

Items	Pre-Test (T1)	Post-Test (T2)	One-Year Follow-Up (T3)	χ^2^	Post Hoc Test
	M ± SD	M ± SD	M ± SD		
I would accept a person with mental illness as a neighbour.	2.93 ± 0.88	2.20 ± 0.56	1.80 ± 0.68	14.93 **	T1 > T3
I would accept a person with mental illness as a co-worker.	3.07 ± 0.62	2.07 ± 0.62	1.79 ± 0.70	19.4 ***	T1 > T2 T1 > T3
I would accept a person with mental illness as a friend.	2.40 ± 0.91	1.60 ± 0.63	1.53 ± 0.74	10.85 **	-
I would accept a person with mental illness to rent my house.	3.33 ± 0.62	2.27 ± 0.80	2.20 ± 0.86	16.18 ***	T1 > T2 T1 > T3
I would recommend a person with mental illness for a job.	2.60 ± 0.91	1.73 ± 0.88	1.73 ± 0.70	12.79 **	T1 > T2 T1 > T3
I would accept a person with mental illness as an in-law.	3.33 ± 1.047	2.93 ± 0.80	2.93 ± 0.96	3.05	-
I would accept a person with mental illness to take care of my child.	4.07 ± 0.88	3.20 ± 1.01	3.27 ± 1.03	8.21 *	-

* p < 0.05, ** *p* < 0.01, *** *p*< 0.001.

**Table 4:** The results of Friedman test on the pre-test, post-test, and follow-up scores for the questionnaires on stigmatising attitudes among students who completed the course (*n* = 15).

Domains	Pre-Test (T1)	Post-Test (T2)	One-Year Follow-Up (T3)	χ^2^	Post Hoc Test
	M ± SD	M ± SD	M ± SD		
Stigmatising attitudes towards mental illness					
Deviant behaviour	3.47 ± 0.95	2.13 ± 0.79	2.51 ± 0.77	13.62 **	T1 > T2 T1 > T3
Social isolation	2.69 ± 0.85	2.04 ± 0.55	1.73 ± 0.34	14.80 **	T1 > T2 T1 > T3
Negative stereotype	2.04 ± 0.68	1.52 ± 0.53	1.77 ± 0.79	6.37 *	-
Self-stigma	2.27 ± 0.81	1.69 ± 0.62	2.11 ± 0.87	3.57	-
Average	2.62 ± 0.61	1.80 ± 0.48	2.00 ± 0.55	11.58 **	T1 > T2
Stigmatising attitudes towards children with emotional behavioural disorders (EBD)					
Rejective attitude	2.41 ± 0.68	2.13 ± 0.35	1.98 ± 0.68	2.18	-
Deviant behaviour	2.95 ± 0.67	2.42 ± 0.68	2.34 ± 0.60	9.72 **	T1 > T3
Negative stereotype	2.89 ± 0.70	2.33 ± 0.60	2.07 ± 0.63	10.33 **	T1 > T3
Average	2.77 ± 0.57	2.31 ± 0.51	2.18 ± 0.57	8.78 *	T1 > T3
Stigmatising attitudes towards disabilities					
Negative stereotype	2.80 ± 1.01	2.51 ± 1.04	2.76 ± 1.02	4.50	-
Pessimistic expectation	2.69 ± 0.89	2.49 ± 0.69	2.51 ± 0.80	1.04	-
Average	2.74 ± 0.88	2.50 ± 0.82	2.63 ± 0.85	4.42	-

* p < 0.05, ** *p* < 0.01, *** *p* < 0.001.

**Table 5:** The results of Mann–Whitney U tests on the pre-test/follow-up change scores between students who completed the course (*n* = 15) and those who dropped the course (*n* = 10).

Domains/Items	Drop			Complete ^1^	*Z*
	Pre-Test	One-Year Follow-Up	Change Scores	Change Scores	
	M ± SD	M ± SD	M ± SD	M ± SD	
Stigmatising attitudes towards					
Mental illness	2.27 ± 0.62	2.16 ± 0.66	−0.11 ± 0.81	−0.61 ± 0.78	−1.73
EBD ^2^	2.52 ± 0.90	2.39 ± 0.71	−0.14 ± 0.92	−0.59 ± 0.75	−1.76
Disability	2.74 ± 0.88	3.22 ± 0.28	−0.10 ± 1.04	−0.11 ± 0.64	1.00
Social Distance Scale					
As a neighbour	2.40 ± 0.70	2.40 ± 0.97	0 ± 1.24	−1.13 ± 0.83	−2.27 *
As a co-worker	2.20 ± 0.79	2.20 ± 0.79	0 ± 1.05	−1.29 ± 0.83	−2.74 **
As a friend	1.90 ± 0.57	2.10 ± 0.88	0.20 ± 0.79	−0.87 ± 1.06	−2.43 *
To rent house	2.50 ± 0.53	2.80 ± 0.92	0.30 ± 0.95	−1.13 ± 0.99	−3.02 **
Recommend job	1.90 ± 0.57	1.80 ± 0.63	−0.10 ± 0.57	−0.87 ± 0.92	−2.22 *
As an in-law	3.10 ± 0.99	3.00 ± 0.94	−0.10 ± 1.10	−0.40 ± 1.06	−0.78
Take care of my child	3.70 ± 0.68	3.40 ± 1.08	−0.30 ± 0.82	−0.80 ± 1.08	−1.34

^1^ See Table 3 and Table 4 for the pre-test and follow-up scores of students who completed the course. ^2^ EBD = emotional behavioural disorders. * *p* < 0.05, ** *p* < 0.01.

## References

[1] Jones N., Corrigan P.W., Corrigan P.W. (2014). Understanding Stigma. The Stigma of Disease and Disability: Understanding Causes and Overcoming Injustices.

[2] Morgan A.J., Reavley N.J., Jorm A.F., Beatson R. (2016). Experiences of discrimination and positive treatment from health professionals: A national survey of adults with mental health problems. Aust. N. Z. J. Psychiatry.

[3] Satchidanand N., Gunukula S.K., Lam W.Y., McGuigan D., New I., Symons A.B., Withiam-Leitch M., Akl E.A. (2012). Attitudes of healthcare students and professionals toward patients with physical disability: A systematic review. Am. J. Phys. Med. Rehabil..

[4] Schulze B., Angermeyer M.C. (2003). Subjective experiences of stigma. a focus group study of schizophrenic patients, their relatives and mental health professionals. Soc. Sci. Med..

[5] Corrigan P. (2004). How stigma interferes with mental health care. Am. Psychol..

[6] Riffel T., Chen S.P. (2019). Exploring the knowledge, attitudes, and behavioural responses of healthcare students towards mental illnesses-a qualitative study. Int. J. Environ. Res. Public Health.

[7] Ay P., Save D., Fidanoglu O. (2006). Does stigma concerning mental disorders differ through medical education? A survey among medical students in Istanbul. Soc. Psychiatry Psychiatr. Epidemiol..

[8] Abbey S., Charbonneau M., Tranulis C., Moss P., Baici W., Dabby L., Gautam M., Paré M. (2011). Stigma and discrimination. Can. J. Psychiat..

[9] Sherwood D.A. (2019). Healthcare curriculum influences on stigma towards mental illness: Core psychiatry course impact on pharmacy, nursing and social work student attitudes. Curr. Pharm. Teach. Learn..

[10] Chang S., Ong H.L., Seow E., Chua B.Y., Abdin E., Samari E., Teh W.L., Chong S.A., Subramaniam M. (2017). Stigma towards mental illness among medical and nursing students in Singapore: A cross-sectional study. BMJ Open.

[11] Patten S.B., Remillard A., Phillips L., Modgill G., Szeto A.C., Kassam A., Gardner D.M. (2012). Effectiveness of contact-based education for reducing mental illness-related stigma in pharmacy students. BMC Med. Educ..

[12] Papish A., Kassam A., Modgill G., Vaz G., Zanussi L., Patten S. (2013). Reducing the stigma of mental illness in undergraduate medical education: A randomized controlled trial. BMC Med. Educ..

[13] Martinez-Martinez C., Sanchez-Martinez V., Sales-Orts R., Dinca A., Richart-Martinez M., Ramos-Pichardo J.D. (2019). Effectiveness of direct contact intervention with people with mental illness to reduce stigma in nursing students. Int. J. Ment. Health Nurs..

[14] Knaak S., Patten S.B. (2014). Building and Delivering Successful Anti-Stigma Programs for Healthcare Providers.

[15] Knaak S., Patten S. (2016). A grounded theory model for reducing stigma in health professionals in Canada. Acta Psychiatr. Scand..

[16] Knaak S., Ungar T., Patten S. (2015). Seeing is believing: Biological information may reduce mental health stigma amongst physicians. Aust. N. Z. J. Psychiatry.

[17] Knaak S., Mantler E., Szeto A. (2017). Mental illness-related stigma in healthcare: Barriers to access and care and evidence-based solutions. Healthc. Manag. Forum.

[18] Mezirow J., Mezirow J. (1990). How Critical Reflection Triggers Transformative Learning. Fostering Critical Reflection in Adulthood.

[19] Sukhera J., Chahine S. (2016). Reducing mental illness stigma through unconscious bias-informed education. MedEdPublish.

[20] Sukhera J., Watling C. (2018). A framework for integrating implicit bias recognition into health professions education. Acad. Med..

[21] Deb T., Lempp H., Bakolis I., Vince T., Waugh W., Henderson C., INDIGO READ Study Group (2019). Responding to experienced and anticipated discrimination (READ): Anti-stigma training for medical students towards patients with mental illness—Study protocol for an international multisite non-randomised controlled study. BMC Med. Educ..

[22] Bamgbade B.A., Barner J.C., Ford K.H. (2017). Evaluating the impact of an anti-stigma intervention on pharmacy students’ willingness to counsel people living with mental illness. Community Ment. Health J..

[23] Radmanović M.B., Burgić S. (2017). Stigma and mental disorders in developmental age. Psychiatr. Danub..

[24] Link B.G., Cullen F.T., Frank J., Wozniak J.F. (1987). The social rejection of former mental patients: Understanding why labels matter. Am. J. Sociol..

[25] Lien Y.J., Kao Y.C. (2019). Public beliefs and attitudes toward schizophrenia and depression in Taiwan: A nationwide survey. Psychiatry Res..

[26] Ma H.I., Hsieh C.E. (2020). Questionnaires on stigmatizing attitudes among healthcare students in Taiwan: Development and validation. BMC Med. Educ..

[27] Ericsson K.A., Simon H.A. (1993). Protocol Analysis: Verbal Reports as Data.

[28] Aronson L. (2011). Twelve tips for teaching reflection at all levels of medical education. Med. Teach..

[29] Siegel S., Castellan N.J. (1988). Nonparametric Statistics for the Behavioural Sciences.

[30] Helmus K., Schaars I.K., Wierenga H., de Glint E., van Os J. (2019). Decreasing stigmatization: Reducing the discrepancy between “us” and “them”: An intervention for mental health care professionals. Front. Psychiatry.

[31] Friedrich B., Evans-Lacko S., London J., Rhydderch D., Henderson C., Thornicroft G. (2013). Anti-stigma training for medical students: The Education Not Discrimination project. Br. J. Psychiatry.

[32] Stier A., Hinshaw S.P. (2007). Explicit and implicit stigma against individuals with mental illness. Aust. Psychol..

[33] Fisher R.J., Katz J.E. (2000). Social-desirability bias and the validity of self-reported values. Psychol. Mark..

[34] Henderson C., Evans-Lacko S., Flach C., Thornicroft G. (2012). Responses to mental health stigma questions: The importance of social desirability and data collection method. Can. J. Psychiatry.

[35] FitzGerald C., Hurst S. (2017). Implicit bias in healthcare professionals: A systematic review. BMC Med. Ethics.

[36] Heim E., Henderson C., Kohrt B.A., Koschorke M., Milenova M., Thornicroft G. (2019). Reducing mental health-related stigma among medical and nursing students in low- and middle-income countries: A systematic review. Epidemiol. Psychiatr. Sci..

[37] Ungar T., Knaak S., Szeto A.C. (2016). Theoretical and Practical Considerations for Combating Mental Illness Stigma in Health Care. Community Ment. Health J..

[38] Ma H.I., Saint-Hilaire M., Thomas C.A., Tickle-Degnen L. (2016). Stigma as a key determinant of health-related quality of life in Parkinson’s disease. Qual. Life Res..

[39] Verhaeghe M., Bracke P. (2012). Associative stigma among mental health professionals: Implications for professional and service user well-being. J. Health Soc. Behav..

[40] Braveman P. (2014). What are health disparities and health equity? We need to be clear. Public Health Rep..

